# The Effects of Tertiary and Quaternary Infections on the Epidemiology of Dengue

**DOI:** 10.1371/journal.pone.0012347

**Published:** 2010-08-23

**Authors:** Paul S. Wikramaratna, Cameron P. Simmons, Sunetra Gupta, Mario Recker

**Affiliations:** 1 Department of Zoology, University of Oxford, Oxford, United Kingdom; 2 Oxford University Clinical Research Unit, Hospital for Tropical Diseases, Ho Chi Minh City, Viet Nam; Global Viral Forecasting Initiative, United States of America

## Abstract

The epidemiology of dengue is characterised by irregular epidemic outbreaks and desynchronised dynamics of its four co-circulating virus serotypes. Whilst infection by one serotype appears to convey life-long protection to homologous infection, it is believed to be a risk factor for severe disease manifestations upon secondary, heterologous infection due to the phenomenon of Antibody-Dependent Enhancement (ADE). Subsequent clinical infections are rarely reported and, since the majority of dengue infections are generally asymptomatic, it is not clear if and to what degree tertiary or quaternary infections contribute to dengue epidemiology. Here we investigate the effect of third and subsequent infections on the transmission dynamics of dengue and show that although the qualitative patterns are largely equivalent, the system more readily exhibits the desynchronised serotype oscillations and multi-annual epidemic outbreaks upon their inclusion. More importantly, permitting third and fourth infections significantly increases the force of infection without resorting to high basic reproductive numbers. Realistic age-prevalent patterns and seroconversion rates are therefore easier reconciled with a low value of dengue's transmission potential if allowing for more than two infections; this should have important consequences for dengue control and intervention measures.

## Introduction

**Figure 1:**
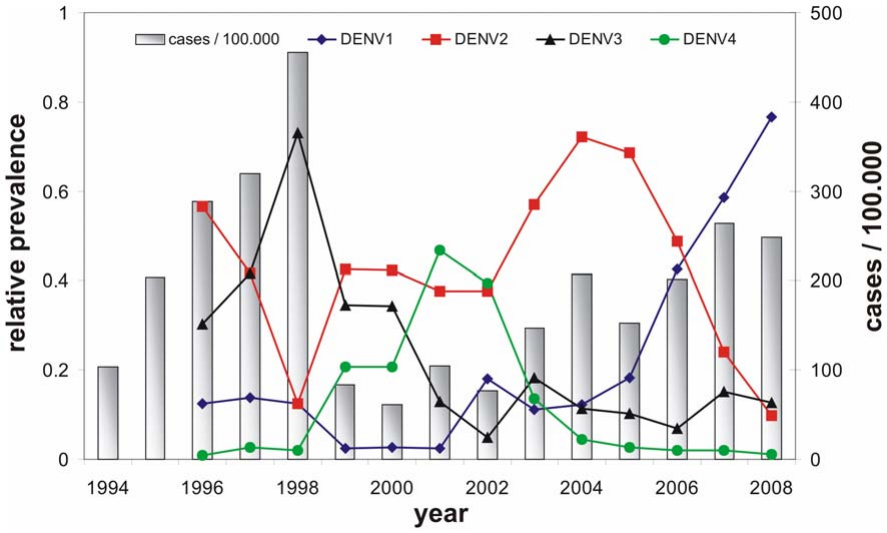
Dengue epidemiology in South Vietnam. The total annual number of dengue cases (blue bars) and relative serotype prevalence (lines) over the period 1994–2008 in the southern 20 provinces of Viet Nam show the characteristic fluctuation in disease incidence and sequential replacements of dominant serotypes. Source of data: Vietnamese Ministry of Health Dengue passive surveillance scheme and kindly provided by the Pasteur Institute, HCMC, Viet Nam. The Hospital for Tropical Diseases is a tertiary referral hospital for infectious diseases.

Dengue viruses belong to the *Flavivirus* group of the family *Flaviviridae* and today represent a major global concern; transmitted from person to person mainly by the mosquito vector *Aedes aegypti* (and to a lesser degree *Aedes albopictus*) they infect roughly 50 million people every year. Of these, some tens of thousands die mostly from the more serious disease forms dengue hemorrhagic fever (DHF) and dengue shock syndrome (DSS). Without treatment, case-fatality rates for the latter can be as high as 20%, though this drops to around 1% with medical intervention. The virus itself is organised into four closely related, co-circulating serotypes: DENV-1, DENV-2, DENV-3 and DENV-4 and long-term epidemiological data reveal multi-annual cycles in disease prevalence and sequential replacement of the dominant serotypes (see Figure 1).

One distinguishing feature of dengue infection is that the risk of developing DHF and DSS is increased by previous exposure. Although infection by one serotype results in an individual gaining complete protective immunity to homologous infection, the immune response stimulated by this exposure paradoxically renders the individual much more likely to develop DHF and DSS upon secondary infection by a heterologous virus. This is believed to be due to the phenomenon of ‘Antibody-Dependent Enhancement’ (ADE) whereby sub-neutralising titres of cross-reactive antibodies promote viral replication [1]–[3].

The mechanism of ADE and its effect on dengue epidemiology have been extensively investigated by both clinical studies and mathematical models (for example [1], [3]–[9]), and it has been established that there is a competitive advantage for serotypes conferring ADE. However, there also is a limit on how large the effect can be before it induces large amplitude oscillations in serotype incidence that could threaten their continued persistence [6]. Furthermore, enhancement of either susceptibility to and/or transmissibility of secondary infection through the action of ADE seems sufficient to explain the desynchronised serotype dynamics and observed 3–5 year epidemic cycles [7], [9]. However, other factors such as temporary or clinical cross-protection with or without seasonal forcing have also been shown to desynchronise the system into irregular epidemic behaviour [8], [10]–[12], and it is not yet clear if dengue epidemiology can really be attributed to any of these factors alone or if it is indeed their combined effect.

A major obstacle in determining the underlying nature of dengue epidemiology lies in the fact that most data are based on clinically reported cases. However, it is widely recognised that a high proportion of dengue infections are asymptomatic and clinically imperceptible [1], [13], [14]. For example, a 1996 study in Haiti demonstrated that over 85% of children had antibodies to two or more dengue serotypes despite no child having been hospitalized or dying with clinical symptoms or signs suggestive of DHF/DSS for at least 16 years [15]. A further complication is a potential bias in much of the available data towards first and secondary infection. This is mainly because of the rarity of clinically observed third and fourth infections [16], [17] but also because of the antibodies' high cross-reactivity that complicates the distinction of more than two preceding infections.

The lack of knowledge in this area is also reflected in the various theoretical approaches to elucidating dengue's intriguing epidemiology: whilst some models explicitly include the possibility of third and fourth infection (e.g. [10], [11], [18]) others have assumed complete immunity after a secondary heterologous infection (e.g. [6], [9]). In this paper we explicitly examine the effect of tertiary and quaternary dengue infections by contrasting the epidemiological dynamics of a previously analysed ‘twice infected – protected’ model [9] to one that permits further infections. We show that whilst preserving the general dynamical behaviour, third and subsequent infections significantly affect the overall force of infection and the ensuing age-dependent incidence rates of DHF/DSS in the population.

## Methods

We present two models to compare the effect of tertiary and quaternary infection on the epidemiology and transmission dynamics of four co-circulating dengue serotypes. The models are based on previously published epidemiological frameworks [5], [6], [9] and differ in their assumptions about acquired immunity. In the first model we assume that recovery from a secondary heterologous infection renders the host completely immune against subsequent challenges. In contrast, the second model assumes that exposure to all four serotypes is necessary for complete protection. We assume that after primary infection secondary and subsequent heterologous infections are enhanced by means of ADE. This case clearly represents the other extreme end in terms of cross-protection and enhancement. However, by analysing both extremes it is possible to interpolate other scenarios where subsequent infections are not enhanced or even contribute less to dengue transmission.

In a previous study we have demonstrated that increased transmission and increased susceptibility can both independently and synergistically explain the desynchronised serotype dynamics and irregular epidemic outbreaks [9]. For simplicity, we here assume that enhancement manifests itself solely in terms of increased onward transmission.

### Mathematical models

Within both frameworks we divide the host population into the following classes: 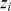 denotes the fraction of the population recovered from infection by at least serotype 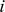; 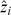 denotes the fraction of the population recovered from infection by serotype 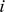 only; 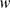 denotes the proportion of the population that has recovered from some form of infection; 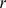 is the proportion of the population completely protected against further infection; 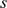 is the proportion of the population completely susceptible to infection; 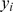 is the proportion infectious with a primary infection with strain 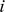; and 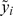 is the fraction infectious with serotype 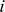 having previously recovered from infection with a heterologous serotype.

The first model, where a secondary infection leads to complete immunity, can then be written as the following set of ordinary differential equations:
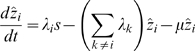


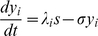


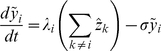


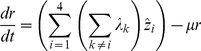
with the proportion susceptible to any infection simply given as 
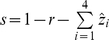
.

The second model, in which immunity is gained only by exposure to all four serotypes, is given as:
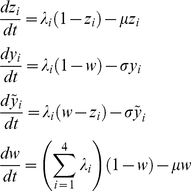
In both models, the host population size is assumed to be constant with 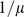 denoting the average host life expectancy and 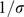 representing the average duration of infection. We have assumed that hosts recover at the same rate from primary and subsequent infections. Since dengue infection generally lasts less than a week [19], we assume the incidence of multiple infections to be negligible.

The force of infection of serotype 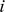, 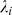, is defined as 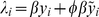, where 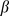 is the transmission coefficient of individuals suffering from a first-time dengue infection. 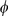 describes the increase in transmissibility during secondary (or subsequent) infections due to the action of antibody dependent enhancement (ADE), with 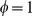 denoting no enhancement and a value of 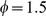, for example, corresponding to a 50% increase in transmissibility.

Although differences in virulence and an effect of infection order on disease outcome have been suggested, we assume there are no differences between the serotypes in terms of transmissibility or duration of infection.

In order to compare and contrast the dynamics of our two models, we performed a series of simulations within the 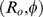 parameter space, with the basic reproduction number 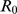 here defined as 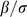. In all cases we varied 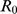 between 2.5 and 4 solely through changes in transmissibility *β*. For simplicity we kept all other parameter values constant and set 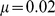, equivalent to an average host life expectancy of 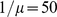 years, and 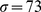, equivalent to an infectious period of 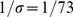 years or ≈5 days. We then explored the effects of both 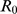 and the level of ADE within our two models.

Unless stated otherwise, all results and qualitative analyses are performed after running our models for sufficient time to remove the effect of transient dynamics and to allow the system to settle onto a particular dynamical behaviour.

## Results

**Figure 2:**
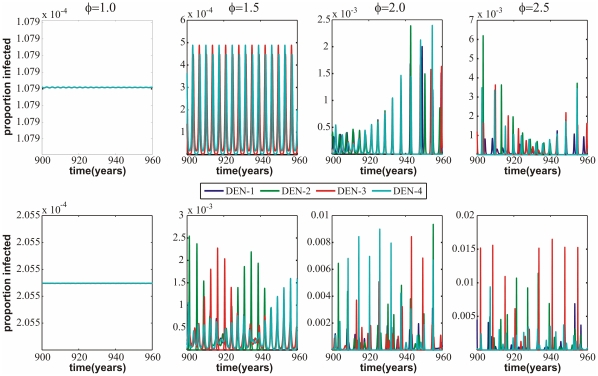
General model dynamics under parameter changes. Here we observe the fluctuations in the total proportion of the population that has been infected by each serotype against time for different levels of transmissibility enhancement 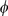 (from left to right: 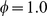, 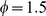, 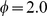, 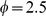; no enhancement is represented by 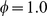; increasing 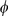 corresponds to augmented levels of enhancement). The top panel is for the model with the assumption of total immunity after two infections only (model (i)) whilst the bottom is for the model which allows for infection with all four serotypes (model (ii)). The individual serotype dynamics seem to become ever more desynchronised as 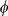 increases in both models, though this effect appears stronger in model (ii).

Figure 2 shows the general model output for fixed 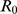 as a selection of time series (after removing the transients) and characterises the wide range of dynamical behaviours observed in both models under increased levels of enhancement 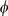. In line with previous studies [5], [6], [9], the following pattern of behaviour emerges. For low levels of enhancement we observe a steady-state equilibrium with all serotypes persisting at equal frequencies. As the degree of enhancement, 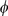, is increased, the equilibrium is replaced by synchronized oscillations. Further augmentation of 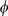 desynchronises these oscillations, leading to the emergence of a more ‘chaotic’ strain structure where individual serotypes are sequentially replaced. Finally, we approach a state where we detect periods of low disease prevalence interspersed with semi-regular epidemic outbreaks. In both models this stage appears to be further characterised by the sequential replacement of the dominant serotype.

Notably, however, the time-series presented in Figure 2 suggest that the progression between these stages is much faster in the model allowing for third and fourth infections and also suggest that the amplitude of oscillations in this case is around 5–10 times larger than in the model where complete protection is attained after only two infections. To qualitatively and quantitatively compare both models and thus highlight the effect of third and fourth infection we used a variety of methods for examining serotype and incidence dynamics.

### Serotype synchronisation

First we analysed the synchronisation pattern between serotypes; we chose serotype 1 and 2 but since the dynamics of each serotype are independent, any pair would give the same results. We distinguished between three different behaviours: (a) complete synchronisation, which indicates that after some initial transient the two serotypes coalesce and remain synchronised for the remainder of a 1000 year time period, that is, the behaviour of one serotype exactly matches that of the other; (b) partial synchronisation, which defines a regime where the serotypes are not fully synchronised but are locked together for at least one period of more than 100 years; and (c) desynchronised for all other cases.

**Figure 3:**
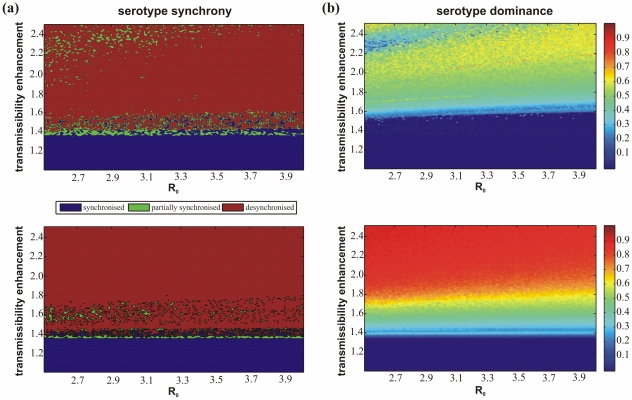
Comparison of synchronisation and single-serotype dominance between model (i) (*top*) and model (ii) (*bottom*). (a) The synchronisation pattern between serotypes 1 and 2 indicates that, for both models, most of parameter space is characterised by desynchronised behaviour i.e. the dynamics of the two serotypes are not ‘locked’ together. (b) Using a measure of single serotype dominance (where 0 corresponds to at least two serotypes being simultaneously dominant and higher values indicate a greater tendency for one serotype to be dominating at any given time), one can observe that in both models the trend is for increasing levels of dominance with increasing enhancement; this trend is more pronounced in model (ii) than model (i).

Figure 3a shows the synchronisation pattern of the two models within the (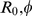)-plane. In both models we notice a general trend from synchronised (blue squares) to desynchronised (red squares) behaviour as we increase the level of enhancement, 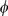. 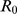, on the other hand, does not seem to have a significant effect on this trend although we find a tendency in model (i) for some partial synchronisation (green squares) at low levels of 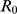 and high levels of 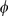, which is not the case in model (ii).

### Sequential replacement of dominant serotypes

We next looked at the serotype dynamics in terms of the sequential replacement of a dominant serotype. To quantify sequential dominance we used the same measure previously introduced by Recker and colleagues [20], given as:
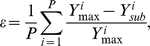
where *P* = number of epidemic peaks observed in a certain time interval considered (here 1000 years), *Y*_max_ = the prevalence of the peaking dominant serotype and *Y*_sub_ = the prevalence of the serotype with the second-highest peak. That is, we analysed the epidemic peaks over a certain time period (here 1000 years) and examined whether and to what degree these epidemics consisted of one or more dengue serotypes, which we averaged across all epidemics within that period. Figure 3b plots 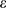 dependent on the level of transmissibility enhancement, 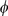, and 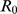. From our definition 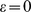 (dark blue in Figure 3b) corresponds to at least two strains always being simultaneously dominant, and 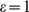 (dark red in Figure 3b) indicates that every epidemic consists of just a single dominant serotype.

Figure 3b demonstrates that in both cases there is a trend of increasing dominance by and thus replacement of a single serotype with increasing levels of enhancement, 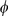, and only a small increase with 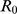. Importantly, this trend is substantially more pronounced when allowing for third and fourth infection in model (ii). That is, sequential replacement of temporary dominant serotypes, which seems characteristic of dengue epidemiology, can be achieved through much smaller levels of enhancement if four infections are required to reach full immunity.

### Inter-epidemic period

**Figure 4:**
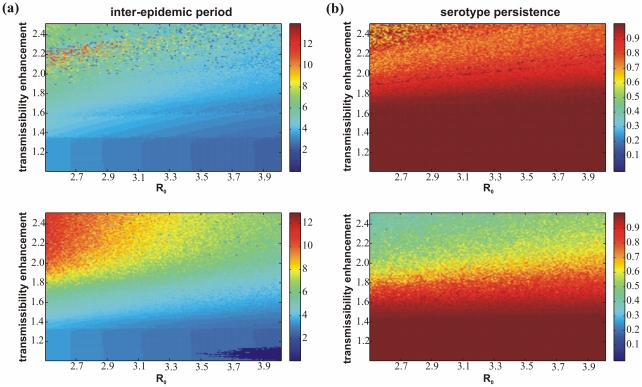
Comparison of inter-epidemic period and serotype persistence between model (i) (*top*) and model (ii) (*bottom*). (a) As enhancement increases so too does the epidemic period observed in each model. There is also a trend towards longer periods at lower 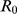. However, these trends both appear to be stronger in model (ii). (b) The risk of stochastic extinction within the model is shown as the proportion of time in each model that the prevalence of a particular serotype exists above a specific threshold. In both models there is a low risk of extinction but the risk increases with enhancement; again, this trend is stronger in model (ii).

We next used standard spectral analysis of the total dengue prevalence to determine the inter-epidemic period under changes in the reproductive number and level of enhancement. Figure 4a plots this period within the (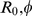)-plane where a blue colouring corresponds to short epidemic cycles of roughly 2 years duration and the transition of the colouring towards red corresponds to a lengthening of the inter-epidemic period. These long periods of low prevalence can generally be interpreted as the result of big epidemic outbreaks that leave the majority of the population immune and require sufficient time for new susceptible to enter the population (usually through birth). Both models readily exhibit the characteristic 3–5 year epidemic cycles of disease incidence associated with dengue for a reasonably wide range of enhancement, 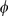. Notably, however, the cycles are generally longer and increase more quickly with increasing levels of enhancement when third and fourth infections are taken into consideration compared to model (i) where immunity is already gained after two infections. We also observe a trend towards longer cycles at high-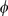, low-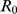; again, this tendency is much less pronounced in model (i). The eventual consequences are cycles in model (ii) that are practically twice as long as those observed in model (i). Indeed, it is very rare in model (i) to observe an inter-epidemic period of longer than 6–7 years whereas in model (ii) a significant region of parameter space is characterised by periods fn the order of 12 years or more.

### Serotype persistence

It has previously been shown that high levels of enhancement increase the risk of serotype extinction due to large epidemic outbreaks leading to extended periods of low transmission [6]. In line with previous work we measured persistence as the proportion of time a particular serotype persists above a given threshold level of 1E-8 [6], [9]. Figure 4b shows that in both models and for low levels of enhancement there is only a marginal risk of stochastic extinction. Nevertheless, and in agreement with previous studies, high levels of enhancement significantly increases this risk of extinction and much more so when allowing for third and fourth infections in model (ii) due to its higher propensity of exhibiting large amplitude oscillations.

### Age structure at equilibrium

**Figure 5:**
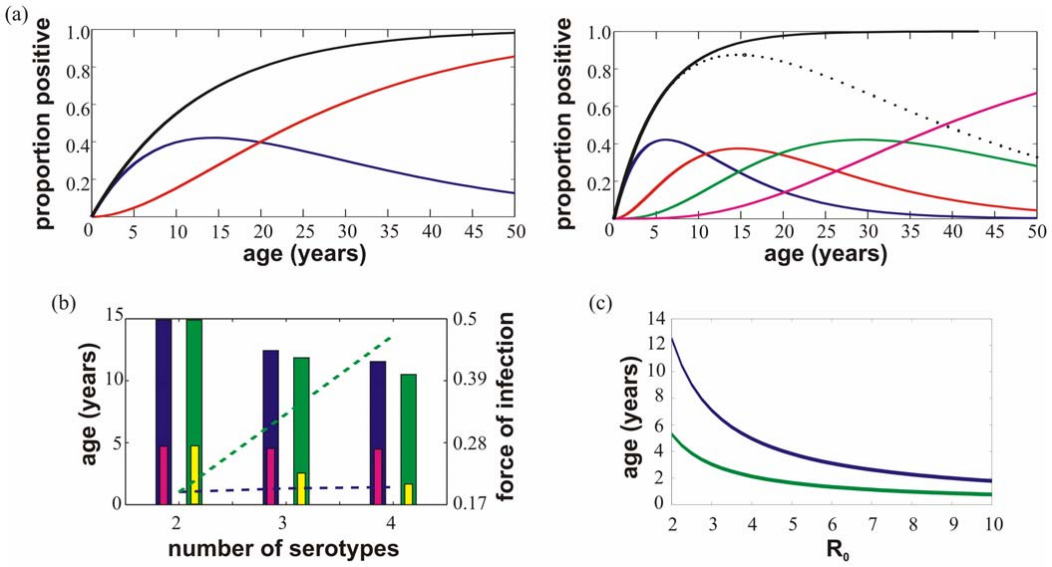
Comparison of age structured dynamics between model (i) and model (ii). (a) The lines show the proportion of the population at each age (for model (i) (top) and model (ii) (bottom)) who have suffered one (solid dark blue line), two (solid red), three (solid green), four (solid magenta) and any (solid black) dengue infections. For model (ii) the proportion of the population that is at risk of disease (defined as having seen 1, 2 or 3 serotypes) is also plotted (dotted black) for comparison to the equivalent in model (i) (solid dark blue). Generally, in model (ii) people are exposed to dengue at an earlier age, experience heterologous infections younger, and take much longer to become completely immune. (b) For model (i) ((ii)), the blue (green) bar shows how the average age of disease (DHF), determined as heterologous infection, changes with the number of serotypes present whilst the small bars show the change in age of first infection. The increase in the total force of infection with the number of serotypes is shown as dotted lines (model (i): blue, and model (ii): green). (c) For model (i) (blue line) and model (ii) (green line) we observe that increasing 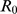 acts to decrease the average age of first infection (here estimated as 1/total force of infection) and that for all levels of 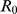 this value is significantly lower when allowing for third and fourth infection (model (ii)). Parameter values: 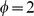 ((a), (b) and (c)) and 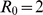 (a), 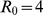 (b).

The only notable effect of third and fourth infections, so far, has been an overall higher propensity for desynchronised, large amplitude oscillations. This, however, can be directly attributed to an overall higher level of transmission that is achieved and maintained by a larger proportion of the population susceptible to infection and onward transmission. We therefore also expect an effect on age-structured prevalence and incidence rates. To explicitly compare the age structure of prevalence within each model we adapted the methods of [21]. We consider in both models the distinct (unstable) equilibrium solutions where all strains have equal forces of infection in the case of medium levels of enhancement (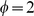). This equilibrium serves as an approximation of the mean prevalence of each serotype over a long period of time. In this case, the proportion of the population that has experienced exactly ‘*i*’ different strains is given by 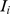. The probability of acquiring a new infection is then proportionate to the number of yet un-encountered strains. In this case with four co-circulating dengue serotypes individuals enter 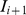 from 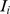 at a rate 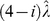, where 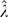 is the average per capita force of infection per strain. The dynamics of this system with respect to time, 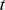, and age, 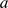, may then be described by the following set of partial differential equations for 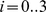:
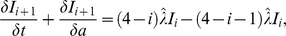
where the proportion of individuals yet unexposed is given by 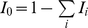. We can then solve these equations (noting, in this instance, that the time derivative is zero) to approximate how the number of infections varies with age in each model. In Figure 5a we have then plotted this data for each model in order to compare and contrast age structures. In Figure 5b we have made a minor adaptation to the models in that we consider the case of just 2 or 3 co-circulating serotypes of dengue. To generate this figure we repeated the above and show how the age of first infection, average age of disease and total force of infection changes for each scenario in both models. Finally, Figure 5c shows how the average age of first infection changes with increasing 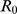 in both of our models.

Figure 5 thus demonstrates that including the possibility of third and fourth infection results in a higher force of infection (Figure 5b), which generally exposes hosts to dengue at an earlier age (see Figure 5c) but also delays the acquisition of full immunity. As an example, it takes roughly twice as long for 50% of the population to become completely immune compared to the case where two infections are sufficient for complete protection. Consequently, allowing for more than two infections increases the pool of people able to onwardly transmit dengue, which in turn explains the observed higher force of infection. We also observe in model (ii) that the proportion of the population that has experienced at least one heterologous infection is greater at all ages, which could translate to an increased risk of DHF/DSS (Figure 5a). Finally, the increased force of infection due to tertiary or quaternary infections marks a much higher level of seroconversion: for example, in model (i), by the age of 10 years, only 30% have seen two infections and 30% are still completely susceptible, whereas by the same age in model (ii) around 60% have seen at least two infections and only 10% remain completely susceptible.

## Discussion

We have constructed a framework to examine the effect of third and subsequent infections on dengue epidemiology. Our results indicate that the qualitative nature of the behaviour observed in models with and without third and fourth infections is predominantly similar – in both models, as we increase the level of antibody dependent enhancement we see a transition from synchronised serotype oscillations to their desynchronisation and an increased tendency towards single serotype dominance and replacement. We also observe an augmented risk of stochastic extinction and longer inter-epidemic periods due to large amplitude epidemic outbreaks that leave the majority of the population immune. Consequently, we can argue that the fundamental properties of both systems remain invariant to the introduction of possible third and fourth infections.

However, there are certain critical quantitative ways in which the results of the models differ from each other. From our analysis, markedly different patterns of the age structure of infections can be observed depending on the number of subsequent infections allowed (Figure 5). A notable feature of dengue is that it can reach very high seroprevalence rates at a relatively early age. For example, a study of 210 6–13 year olds in Haiti revealed that 98% of the cohort had been previously exposed to dengue [15]; similarly, a study of 4–16 year old Nicaraguans found an overall seroprevalence of 91%, with 80% of the children exposed by age 5 [22]. Our results show that in order to obtain seroprevalence rates which are in line with epidemiological data one has to assume values of the basic reproductive number, 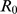, that are very much higher in a ‘twice infected - protected’ framework than if allowing the possibility of a third or fourth infection (Figure 5c). This is because relaxing the assumption that two heterologous infections are sufficient to achieve protection from further challenges leads to an overall higher force of infection, which in turn causes a significant drop in the age of first infection and thus higher levels of seroprevalence across all ages (Figure 5a).

Estimates of the basic reproductive number range from 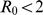 to 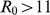, depending on location and the methods used (see e.g. [11], [23]–[26]). However, there are a number of reasons to believe why high values of 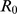 are unlikely for dengue. Many other vector borne pathogens have evolved sophisticated immune evasion mechanisms to prolong their infectious period to overcome the uncertainty in transmission and vector abundance; dengue, by contrast, has a relatively short period of infection [27]. Furthermore, the average lifespan and dispersal pattern of its principle vector, *A. aegypti*, are limited [28], [29], which further reduces the likelihood of sustained transmission but on the other hand necessitates a continuous and large pool of susceptible individuals. Therefore, tertiary and quaternary infections might be critical for reconciling the features of dengue transmission with the observed high levels of transmission and consequently high seroprevalence rates in young children.

As a corollary to the above, increasing the number of co-circulating serotypes has a much greater effect on both the force of infection and age of first infection when allowing for tertiary and quaternary infections. As shown in Figure 5b, and in line with previous work [30], the overall risk of infection in this case increases linearly with the number of serotypes whereas the average age at first infection declines in inverse proportion. By contrast, increasing the number of serotypes has a negligible effect on overall force of infection when assuming full protection after two infections only.

Another important aspect of dengue transmission and 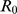 which we have not considered in this work is the effect of spatial heterogeneity and the role of human movement (see e.g. [31], [32]). Although our results are based on simple mass-action principles (i.e. random mixing between individuals) we expect the same to hold true when considering explicit spatial or contact structures. In fact, the possible constraints on dengue transmission imposed by spatial structure should benefit from high levels of third and subsequent infections.

Increasing the number of possible infections and thus the pool of susceptibles also acts to decrease the average age of clinical disease through heterologous re-infections; however, this drop could be partially compensated for by the possibility of developing DHF/DSS also at third and fourth infection. This means that a low age of seroconversion and a high age of DHF/DSS are easier to reconcile if we relax the assumption of clinical protection after secondary infection. The average age of presentation with DHF has seen a steady increase over the last few decades in some parts of SE Asia [33], [34], possibly in response to reduction in transmission through vector control programs [11] or as a consequence of demographic transitions [35]. Our analysis would imply that this is less likely to correlate with an overall drop in seroprevalence if complete cross-protection takes more than two infections to be established.

The two models analysed in this work represent the two extreme ends of a cross-immunity spectrum where at one end two infections are sufficient to protect from further infections and on the other hand where all four serotypes have to be experienced and, importantly, where third and fourth infections are enhanced by pre-existing, cross-reactive antibodies. One can equally imagine the scenario where only secondary infections are enhanced and/or subsequent infections contribute less to dengue transmission through reduced levels of viraemia. However, from our analysis it is clear that in each scenario there will be a direct relationship between the overall level of transmission, force of infection and the resulting age patterns in seroprevalence and incidence of clinical disease.

In conclusion, our results demonstrate that whether or not a ‘twice infected - protected’ hypothesis is a realistic and appropriate description of the full system cannot be determined from the observed epidemiology of dengue and its four serotypes alone. However, allowing more than two infections has a significant impact on the overall force of infection of dengue and could be a more parsimonious explanation for the observed epidemiology than inducing high transmission rates. Crucially also, the fact that low values of 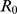 are perfectly compatible with high seroconversion rates is an important consideration for possible future vaccine strategies. On the other hand, though, if third and fourth infections do have a significant contribution to the overall level of dengue transmission, any control strategies based on drug treatment of clinical cases alone can only be expected to have a minor effect. More data on the possibility and transmissibility of tertiary and subsequent infections is therefore of major importance.
